# Supplementary figures and images for: Prognostic implications of cell division cycle protein 45 expression in hepatocellular carcinoma
Source: PeerJ. 2021 Feb 12;9:e10824. doi: 10.7717/peerj.10824 (PMC7883691; doi:10.7717/peerj.10824)

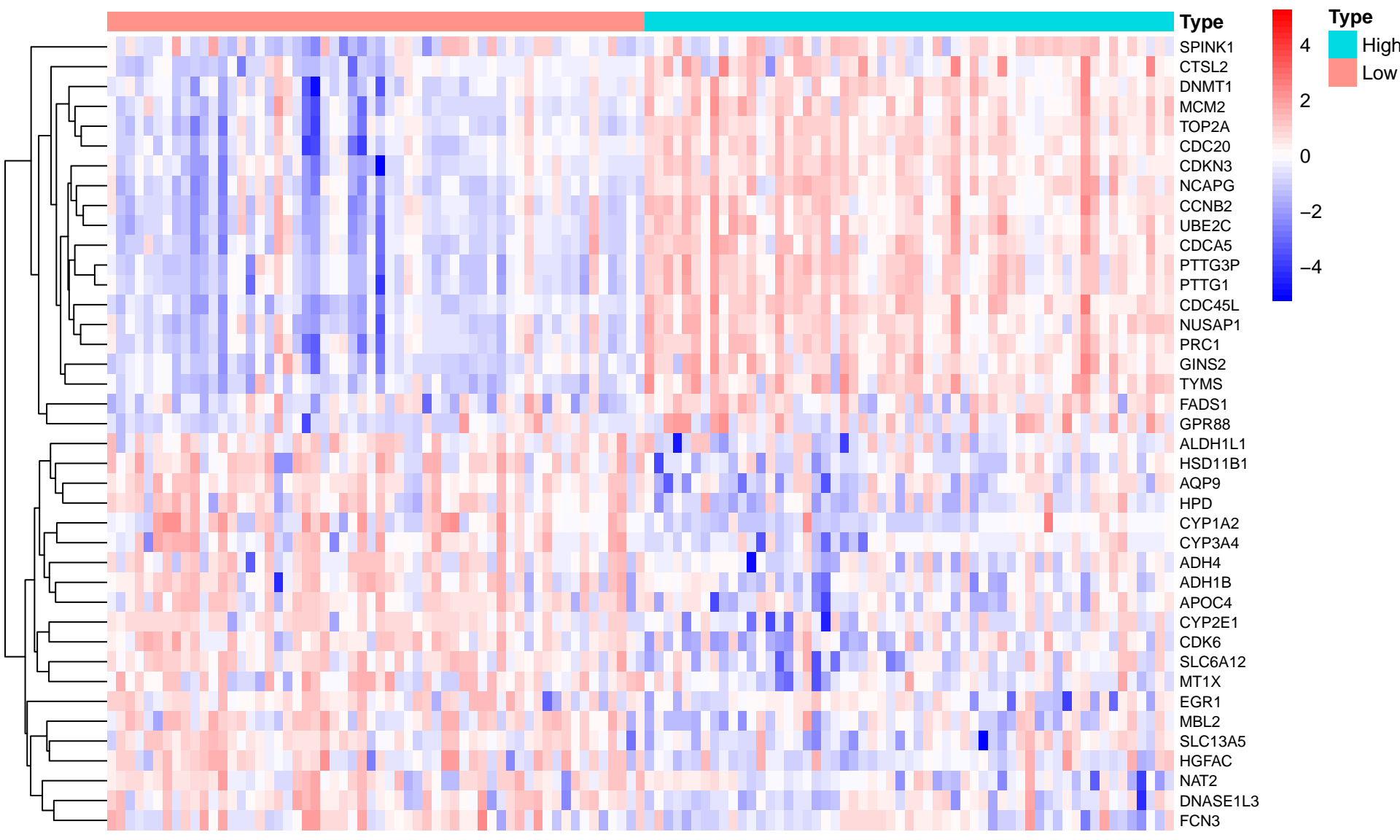

Supplement: File S5 [file peerj-09-10824-s005.zip › Supplement 5.1 File/1.diff/heatmap.pdf]

# Volcano

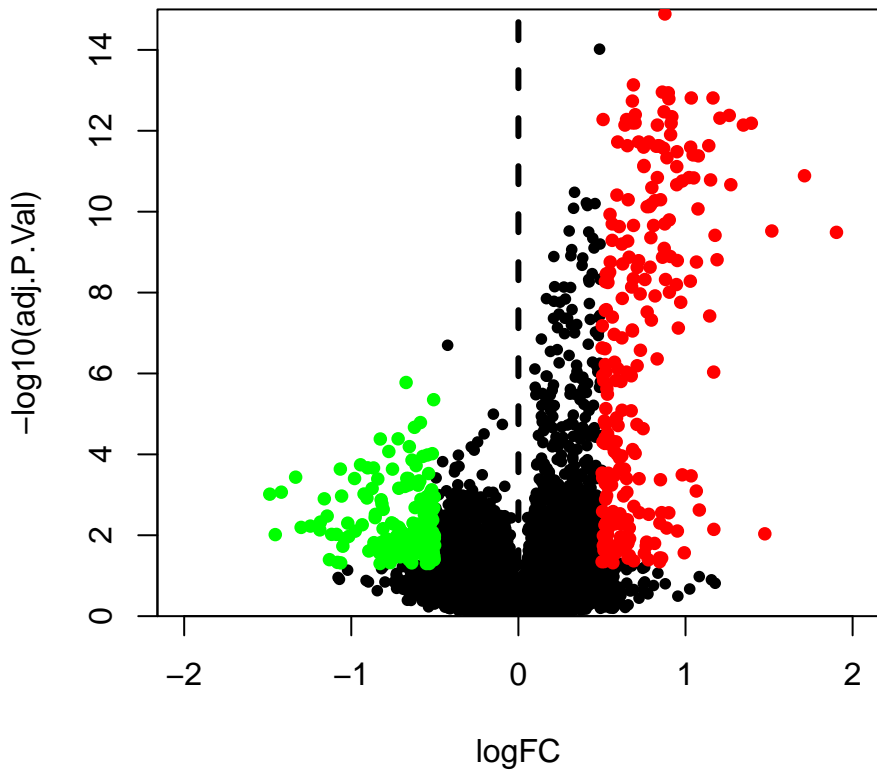

Supplement: File S5 [file peerj-09-10824-s005.zip › Supplement 5.1 File/1.diff/vol.pdf]

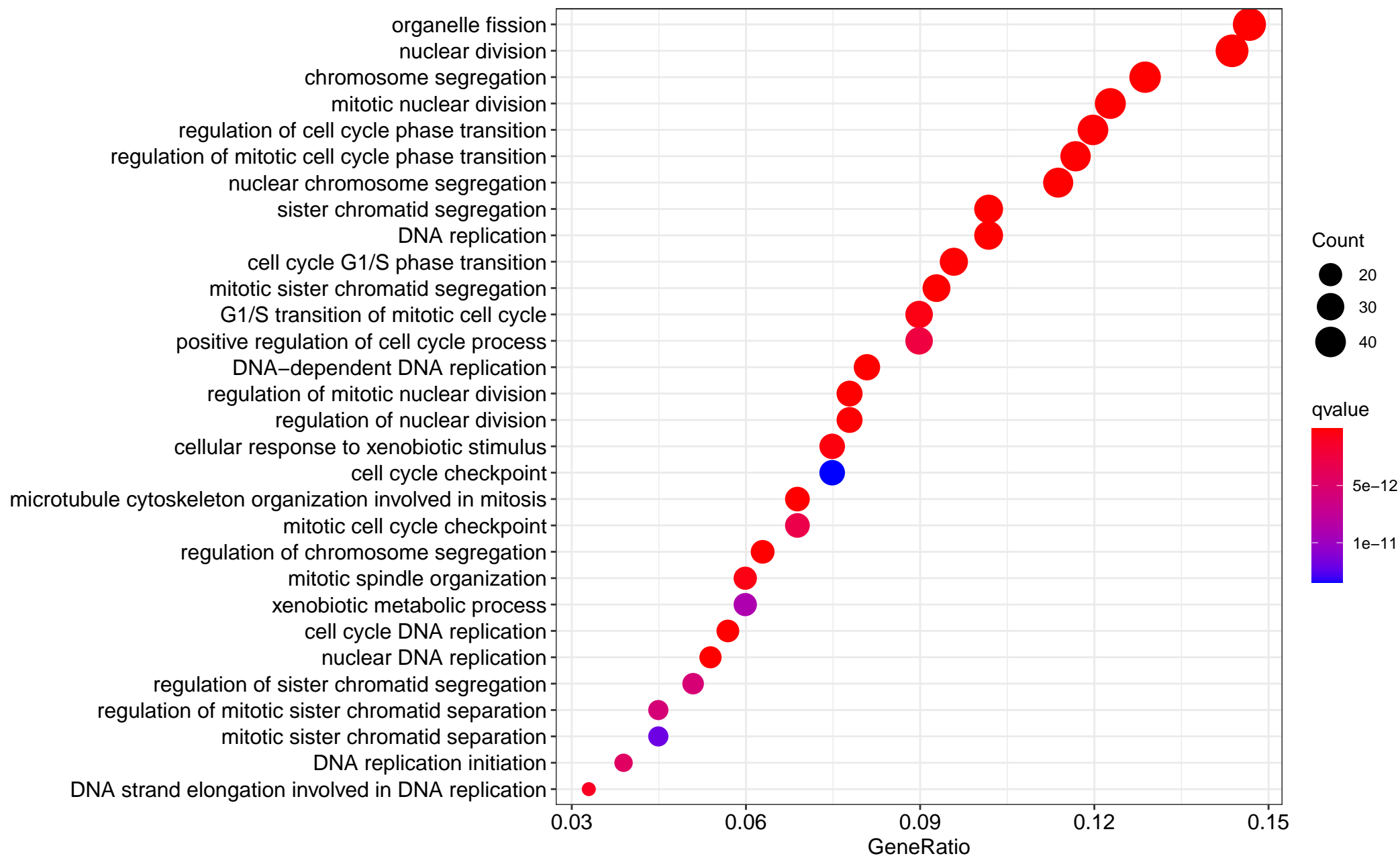

Supplement: Supplemental Information 6 [file peerj-09-10824-s006.zip › Supplement 5.2 File/3.GO/bubble.pdf]

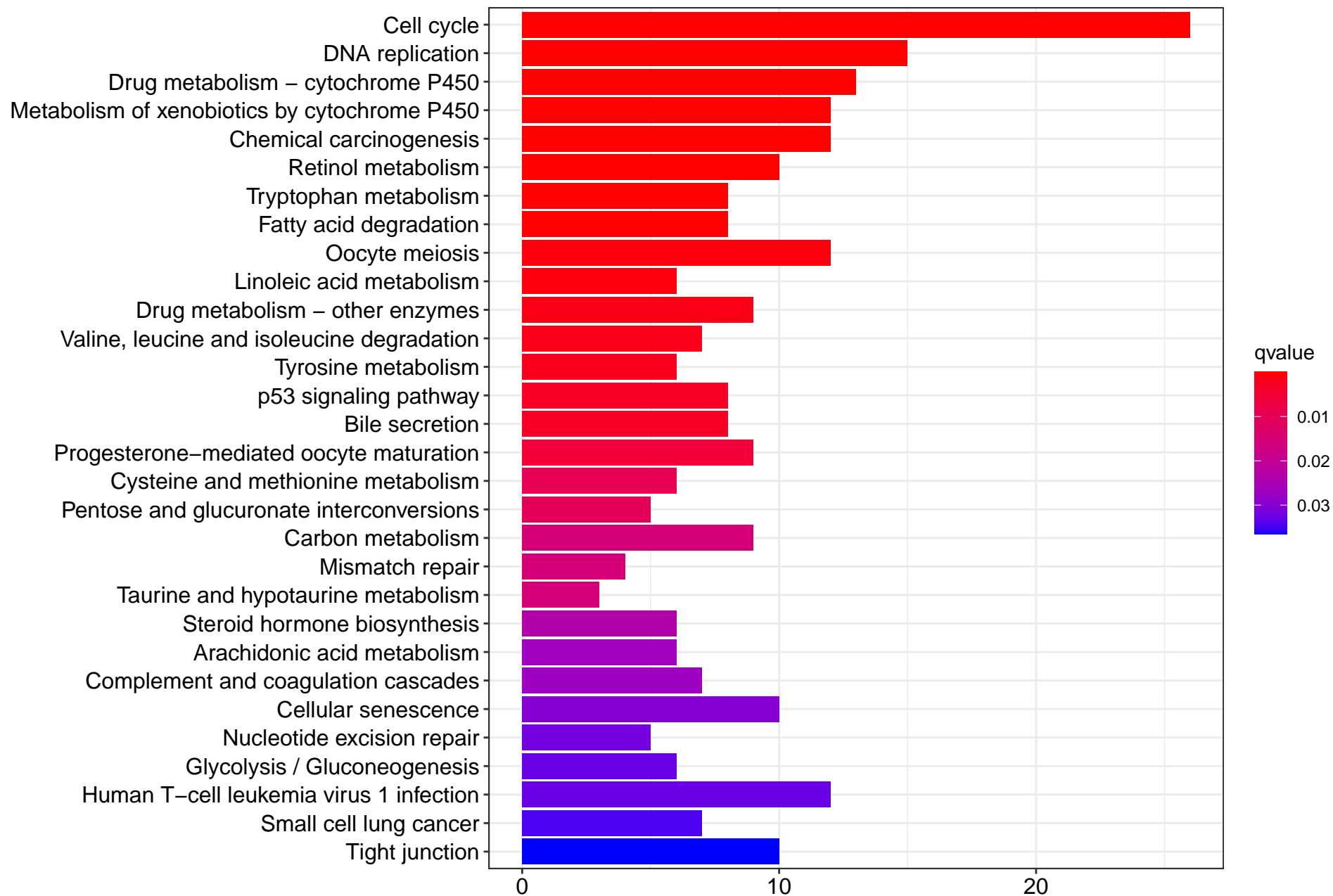

Supplement: Supplemental Information 6 [file peerj-09-10824-s006.zip › Supplement 5.2 File/4.KEGG/barplot.pdf]

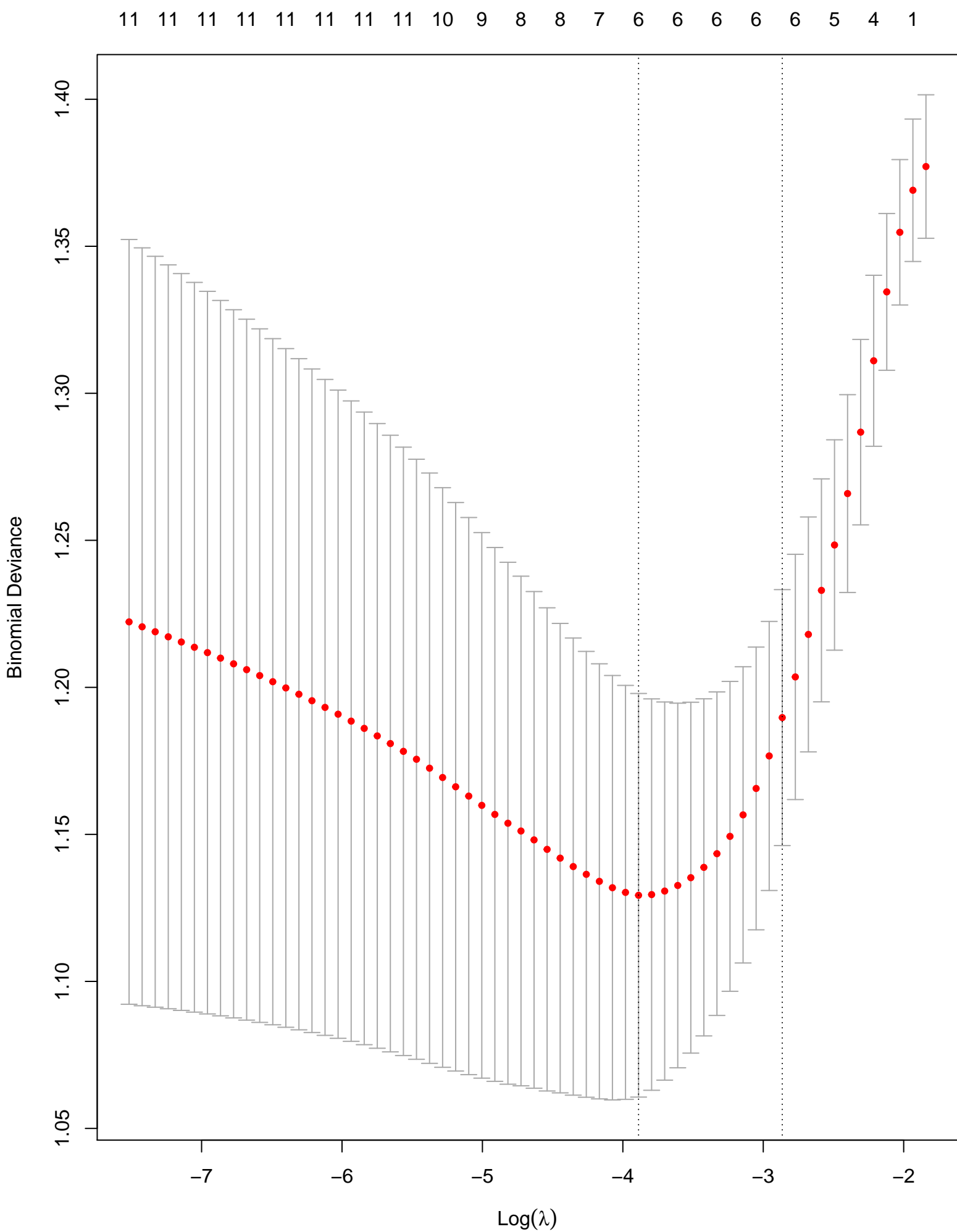

Supplement: File S7 [file peerj-09-10824-s009.zip › supplement 7 File/logλ of Lasso regression.pdf]

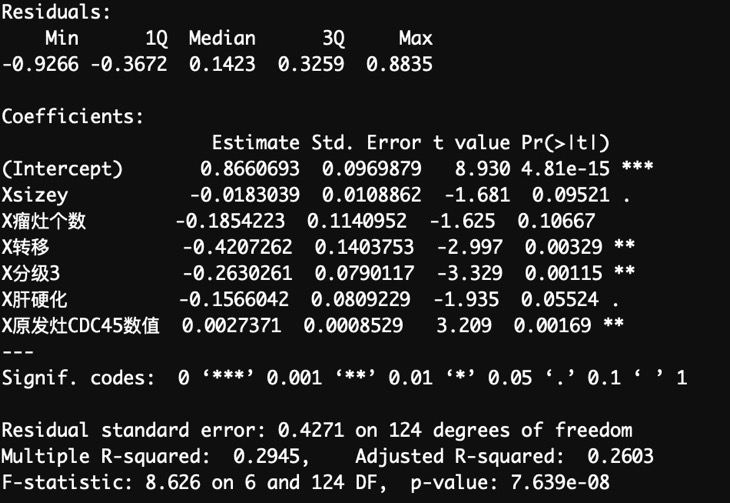

Supplement: File S7 [file peerj-09-10824-s009.zip › supplement 7 File/result of lasso regression.jpg]

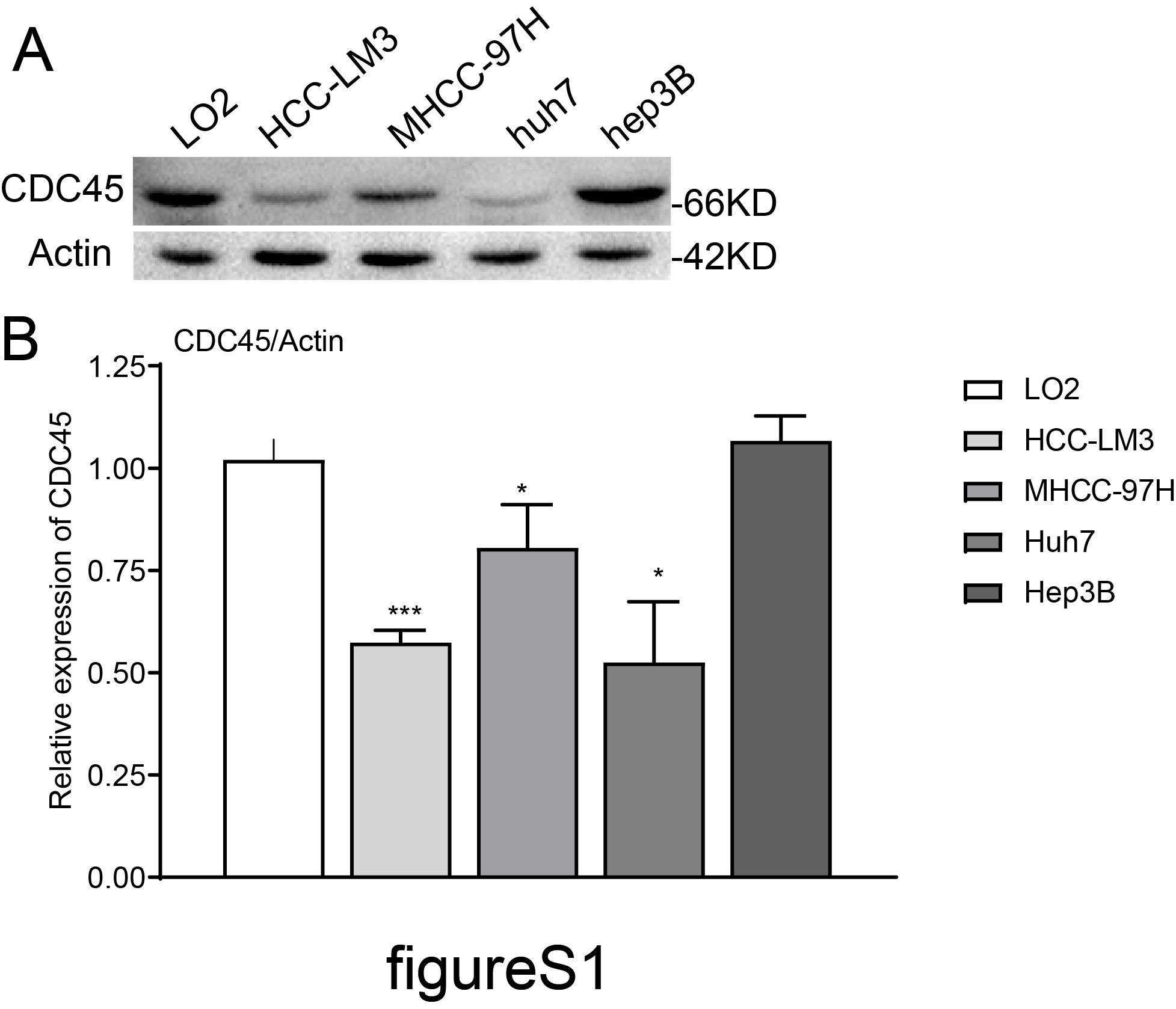

Supplement: File S8 [file peerj-09-10824-s010.zip › Supplement 8 File/Figure S1.png]

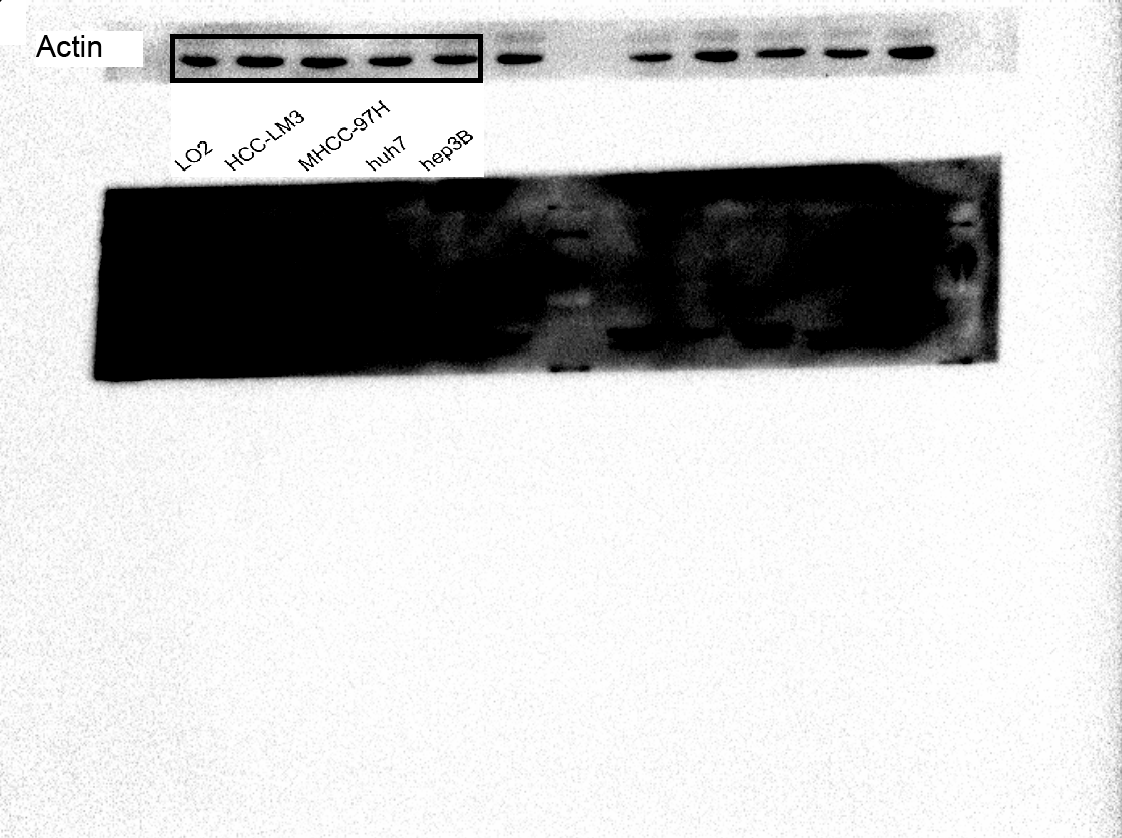

Supplement: File S8 [file peerj-09-10824-s010.zip › Supplement 8 File/oringinal pictures of WB/Actin.tif]

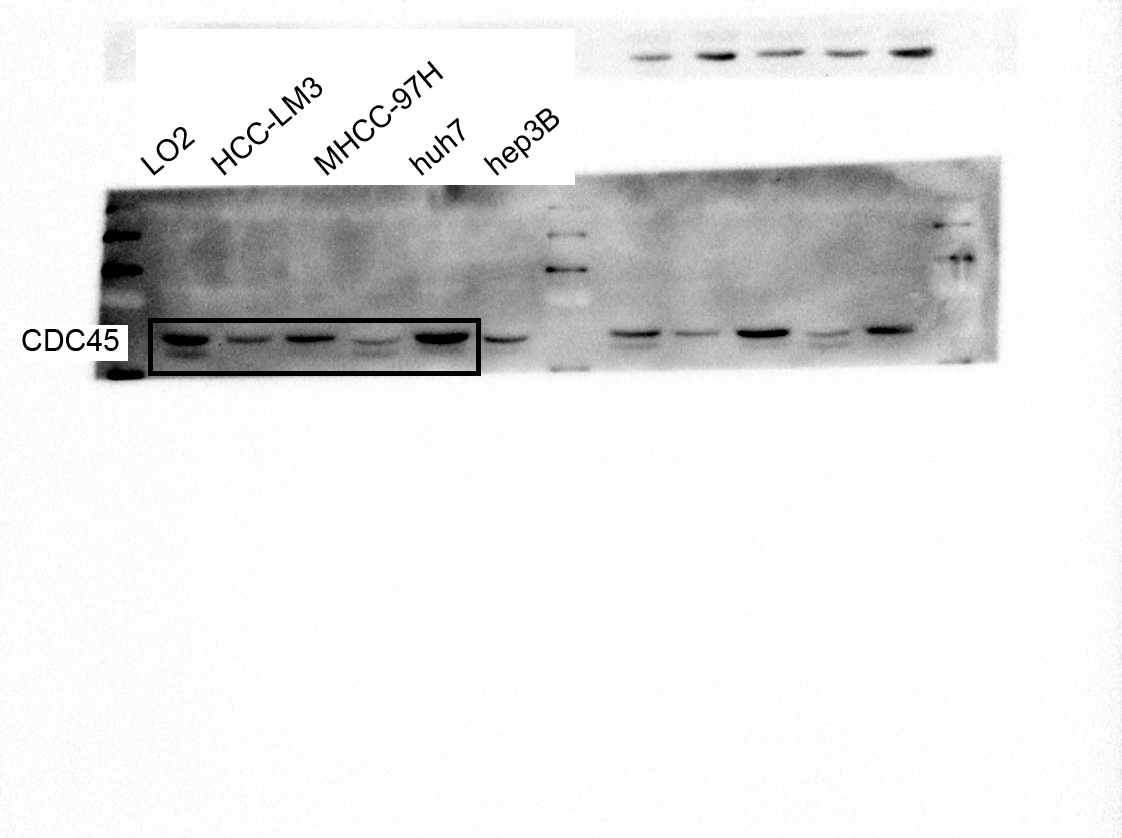

Supplement: File S8 [file peerj-09-10824-s010.zip › Supplement 8 File/oringinal pictures of WB/CDC45.tif]

CDC45L level + high + low

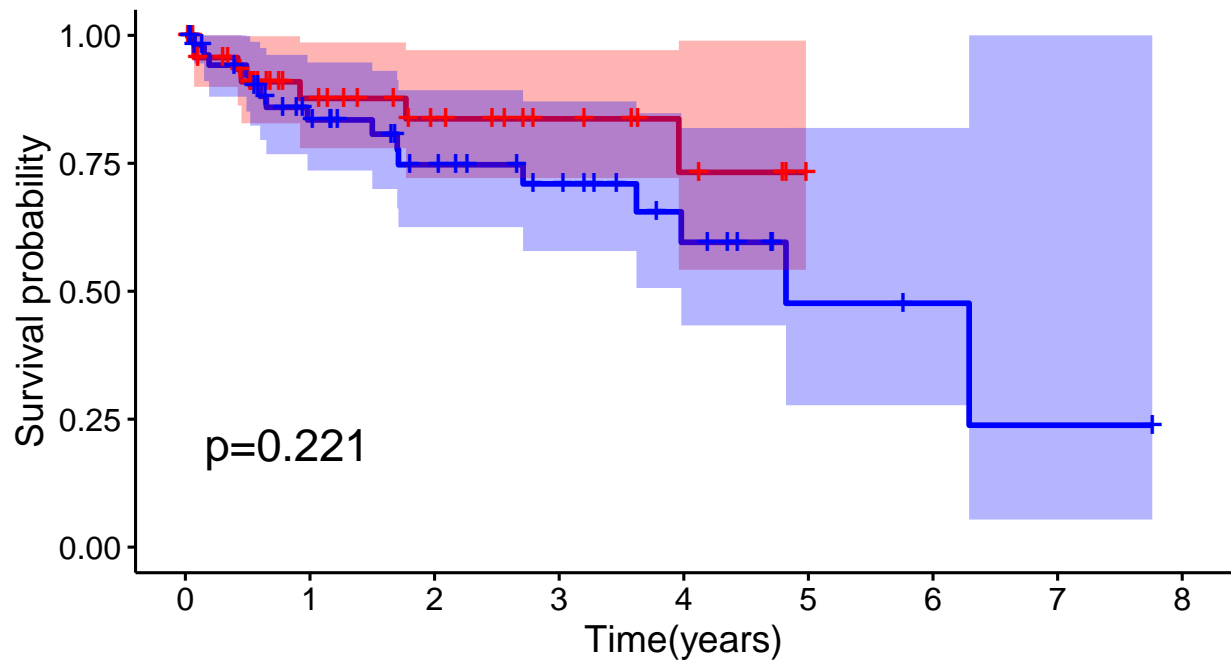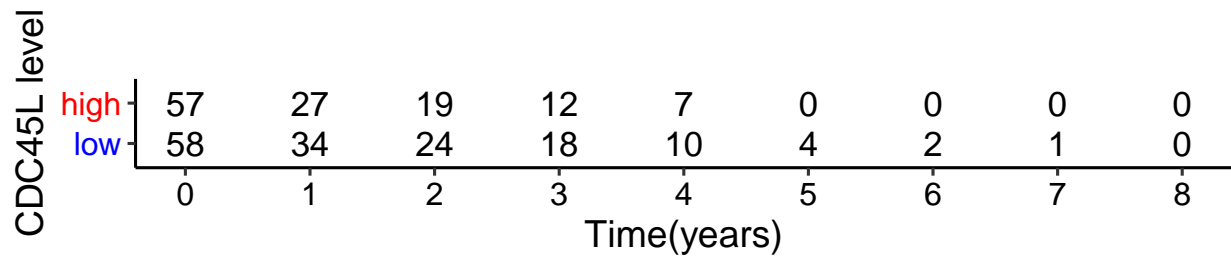

Supplement: File S9 [file peerj-09-10824-s011.zip › Supplement 9 File/CDC45.survival.pdf]
