# Supplementary material for: Prognostic implications of cell division cycle protein 45 expression in hepatocellular carcinoma
Source: PeerJ. 2021 Feb 12;9:e10824. doi: 10.7717/peerj.10824 (PMC7883691; doi:10.7717/peerj.10824)
Supplement: File S8 [file peerj-09-10824-s010.zip › Supplement 8 File/The explanation of Figure S1.docx]

**Cell culture**

Human normal liver cell line LO2 and hepatocellular carcinoma cell lines (HCC-LM3, MHCC-97H, Huh7 and Hep3b) were purchased from the ATCC and cultured in Dulbecco’s modified Eagle’s medium (DMEM, Gibco, USA)) containing 10% fetal bovine serum (Gibco, USA) and 1% penicillin/streptomycin (Beyotime, China) in a humid atmosphere with 5% CO 2 at 37 °C.

**Western blot and antibodies**

Cells were washed with PBS (Beyotime, China) twice. Next, RIPA buffer were used to lyse the cells. Thereafter, BCA Protein Quantitation Assay (Beyotime, China) was used to detected the protein concentration of the cells. Proteins were resolved using 10% SDS-PAGE and then transferred onto a nitrocellulose membrane. After block with 5% non-fat milk for 1h at room temperature, the membranes were then incubated with the primary antibodies at 4°C overnight. After that, TBST was used to wash the membranes three times and subsequently incubated with secondary antibodies (anti-rabbit IgG or anti-mouse IgG) for 1h at room temperature. Next, membranes were washed with TBST three times. The targeted proteins were identified using the ECL (Thermofisher, USA) method. CDC45 and ACTIN antibodies used in study were purchased from HuaBio (ET1701-71, China) and Abcam (ab8227).

**Statistical analyses**

Data were expressed as means±SD. Student’s t-test were used for comparison among groups. P-value less than 0.05 was considered to be significant. Statistical analyses were analysed using GraphPad prism 8.1.

**Results**

Taking into account the reviewer's opinion, we detected the level of CDC45 among human normal liver LO2 cells and a variety of HCC cells. The results showed that CDC45 expression was lower in HCC-LM3 cells, MHCC-97H and Huh7 cells than LO2 cells.

**Figure legend**

Fig S1. Expression of CDC45 in various cell lines (A)Western blot detected the expression of CDC45 in normal human hepatocytes and various hepatoma cell lines. (B) Quantitative analysis of three test results (Values represented mean ± SD.* P < 0.05, *** P < 0.001 versus control)
